# HPV16-Related Cervical Cancers and Precancers Have Increased Levels of Host Cell DNA Methylation in Women Living with HIV

**DOI:** 10.3390/ijms19113297

**Published:** 2018-10-23

**Authors:** Wieke W. Kremer, Marjolein van Zummeren, Daniëlle A. M. Heideman, Birgit I. Lissenberg-Witte, Peter J. F. Snijders, Renske D. M. Steenbergen, Greta Dreyer, Chris J. L. M. Meijer

**Affiliations:** 1 Department of Pathology, Cancer Center Amsterdam, Amsterdam UMC, Vrije Universiteit Amsterdam, 1081 HV Amsterdam, The Netherlands; w.kremer@vumc.nl (W.W.K.); ma.vanzummeren@vumc.nl (M.v.Z.); dam.heideman@vumc.nl (D.A.M.H.); r.steenbergen@vumc.nl (R.D.M.S.); 2 Department of Epidemiology and Biostatistics, Amsterdam Public Health, Amsterdam UMC, Vrije Universiteit Amsterdam, 1081 HV Amsterdam, The Netherlands; b.witte@vumc.nl; 3 Department of Obstetrics and Gynaecology, University of Pretoria, Pretoria 0002, South Africa; gretadreyer@mweb.co.za

**Keywords:** human immunodeficiency virus, human papillomavirus, high-grade cervical intraepithelial neoplasia, DNA methylation, uterine cervical neoplasms

## Abstract

Data on human papillomavirus (HPV) type-specific cervical cancer risk in women living with human immunodeficiency virus (WLHIV) are needed to understand HPV–HIV interaction and to inform prevention programs for this population. We assessed high-risk HPV type-specific prevalence in cervical samples from 463 WLHIV from South Africa with different underlying, histologically confirmed stages of cervical disease. Secondly, we investigated DNA hypermethylation of host cell genes *ASCL1*, *LHX8*, and *ST6GALNAC5*, as markers of advanced cervical disease, in relation to type-specific HPV infection. Overall, HPV prevalence was 56% and positivity increased with severity of cervical disease: from 28.0% in cervical intraepithelial neoplasia (CIN) grade 1 or less (≤CIN1) to 100% in invasive cervical cancer (ICC). HPV16 was the most prevalent type, accounting for 9.9% of HPV-positive ≤CIN1, 14.3% of CIN2, 31.7% of CIN3, and 45.5% of ICC. HPV16 was significantly more associated with ICC and CIN3 than with ≤CIN1 (adjusted for age, OR_MH_ 7.36 (95% CI 2.33–23.21) and 4.37 (95% CI 1.81–10.58), respectively), as opposed to non-16 high-risk HPV types. Methylation levels of *ASCL1*, *LHX8*, and *ST6GALNAC5* in cervical scrapes of women with CIN3 or worse (CIN3+) associated with HPV16 were significantly higher compared with methylation levels in cervical scrapes of women with CIN3+ associated with non-16 high-risk HPV types (*p*-values 0.017, 0.019, and 0.026, respectively). When CIN3 and ICC were analysed separately, the same trend was observed, but the differences were not significant. Our results confirm the key role that HPV16 plays in uterine cervix carcinogenesis, and suggest that the evaluation of host cell gene methylation levels may monitor the progression of cervical neoplasms also in WLHIV.

## 1. Introduction

Human papillomavirus (HPV) is one of the most common sexually transmitted infections, with a worldwide prevalence of approximately 10% in women [1]. Although most infections are asymptomatic and self-limiting, HPV has been widely recognised as the main etiologic agent in cervical cancer development [2,3,4]. Of the more than 200 different types of HPV that have been identified, 14 HPV types have been classified as carcinogenic (16, 18, 31, 33, 35, 39, 45, 51, 52, 56, 58, 59, and 66, International Agency for Research on Cancer (IARC) Group 1) or probably carcinogenic (68, IARC Group 2A) [5]. These high-risk types differ greatly in their oncogenic potential: HPV16 is the most virulent, causing more than 60% of all cervical carcinomas, followed by HPV18 and HPV45, accounting for another 10% and 5%, respectively [6,7].

It has been suggested that the oncogenic potential of high-risk genital HPV types may be influenced by a concomitant infection with the human immunodeficiency virus (HIV) [8,9,10]. HIV positivity and HIV-related immunodeficiency, reflected by low CD4+ T-cell count, are known to be associated with high prevalence and persistence of all high-risk HPV types [11,12], leading to an increased risk of cervical intraepithelial neoplasia (CIN) and invasive cervical cancer in women living with HIV (WLHIV) [13,14,15,16,17]. The proportion of HPV16-related CIN and cervical carcinomas is lower in WLHIV, and non-16 high-risk types are over-represented in this group [18,19]. This difference has been explained by the relative independence of HPV16 infection from immune status, as opposed to other high-risk types, suggesting a strong capacity of HPV16 to evade even normal immune surveillance [8,9,13].

Manipulation of the host cell DNA methylation machinery is known to be an important mechanism by which HPV influences cellular and viral gene expression and is likely one of the mechanisms by which HPV evades antiviral immunity [20]. DNA methylation is a potent epigenetic regulator of gene expression that involves the covalent binding of a methyl group at the carbon-5 position of cytosine located at the 5′ end of a guanine to generate a 5-methylcytosine. Hypermethylation of promoter regions of tumour suppressor genes, resulting from the upregulation of DNA methyltransferase (DNMT) expression caused by the viral oncogenes E6 and E7, leads to gene silencing and is known to be involved in cervical carcinogenesis [21,22,23]. Some methylation patterns have been described to be HPV type-specific [24,25], which may reflect a capacity of specific HPV types to hijack the methylation machinery of the host.

In the present report, we describe HPV type-specific prevalence in cervical samples from WLHIV from South Africa with different underlying, histologically confirmed stages of cervical disease, and explore a possible association with hypermethylation of host cell genes *ASCL1*, *LHX8*, and *ST6GALNAC5*. These genes were identified in a recent genome-wide methylation profiling study on HPV-positive self-collected cervico-vaginal specimens [26]. While these genes have been described as triage markers for HPV-positive women, the genes were also shown to be useful as primary screening markers in WLHIV [27]. To further position these genes as screening or diagnostic markers in WLHIV, additional insight into the relationship between HPV type distribution and methylation patterns in different stages of cervical carcinogenesis of WLHIV is warranted.

## 2. Results

### 2.1. Study Population

**Figure 1:**
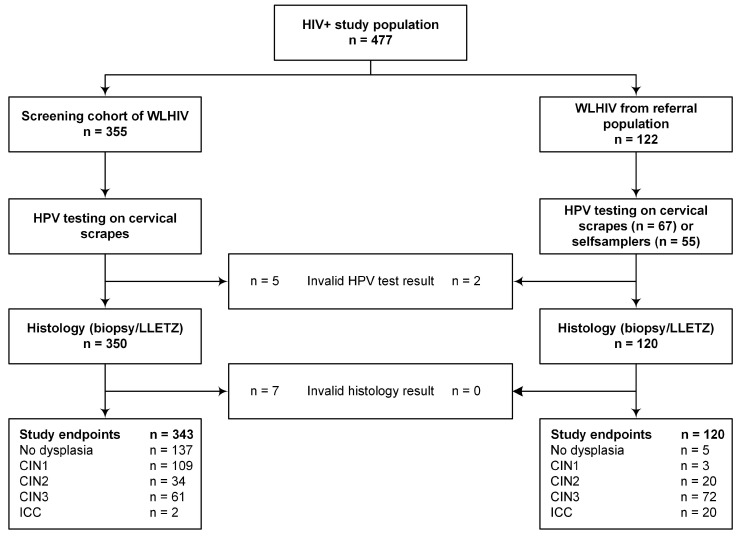
Flowchart. WLHIV, women living with HIV; HPV, human papillomavirus; LLETZ, large loop excision of the transformation zone; CIN, cervical intraepithelial neoplasia; ICC, invasive cervical cancer.

As shown in Figure 1, a total of 463 HIV seropositive women with valid HPV test results and valid histology endpoints were included in the analyses, of whom 142 had no dysplasia, 112 had CIN1, 54 had CIN2, 133 had CIN3, and 22 had invasive cervical cancer (ICC, 19 squamous cell carcinoma, two adenocarcinoma and one unspecified carcinoma). The majority of women (86.0%) were on antiretroviral treatment (ART) at the time of inclusion. The recorded median CD4+ count was 486 cells per microliter (interquartile range (IQR): 312–677 cells per microliter).

### 2.2. HPV Prevalence: Overall and Type-Specific

HPV prevalence within disease categories is shown in Table 1. In total, 258 women (55.7%) tested high-risk HPV positive, of whom 78 (30.2%) had multiple HPV infections. HPV prevalence, including both single and multiple infections, increased with lesion severity. Multiple infections were most frequently observed in women with CIN2, who were the youngest on average (mean age 36.5 years, standard deviation (SD) 7.9), and were least frequently observed in women with ICC, who were the oldest on average (mean age 46.2 years, SD 13.1).

Table 2 displays the HPV type-specific prevalence in HPV-positive women across disease categories—overall and for single infections separately. Overall, HPV16 was the most prevalent type in this study group, present in 24.0% of all HPV-positive cervical samples, followed by HPV52 and HPV45, present in 14.0% and 12.8% of all HPV-positive cervical samples, respectively. HPV16 positivity increased with severity of the cervical lesion, from 9.9% in ≤CIN1, through 14.3% and 31.7% in CIN2 and CIN3, respectively, to 45.5% in ICC; a more or less stable prevalence over disease categories was seen for other types, including HPV18 and HPV45. Disease associations did not change substantially when women with single infections were analysed separately.

HPV was detected in 28.0% of ≤CIN1 and most of these lesions had a single-type infection (77.5%). Almost all HPV types tested were represented in this group. HPV52 was the most prevalent type, accounting for 16.9% of HPV-positive ≤CIN1. HPV18 and HPV45 accounted for another 11.3% and 14.1%, respectively.

HPV was detected in 77.8% of CIN2 lesions and almost half of these had multiple types (48.8%). HPV35 was the most prevalent type, accounting for 21.4% of HPV-positive CIN2 lesions. HPV18 and HPV45 accounted for another 19.0% and 14.3%, respectively.

HPV was detected in 92.5% of CIN3 lesions and most of these lesions had a single-type infection (67.5%). HPV16 was the most prevalent type, accounting for 31.7% of HPV-positive CIN3 lesions. HPV18 and HPV45 were each present in 13.6% of HPV-positive CIN3 lesions. HPV16, -33, -35, -51, -58, and -66 were found more frequently in CIN3 than in ≤CIN1 (CIN3:≤CIN1 ratios 3.22, 1.62, 1.35, 2.89, 2.69, and 2.02, respectively), but for HPV66 this was not observed when only single infections were considered. When adjusted for age, women with CIN3 were significantly more likely to carry HPV16 than women with ≤CIN1 (OR_MH_ 4.37; 95% CI 1.81–10.58); this was also true when only single infections were taken into account.

All ICC tested positive for HPV. The majority were associated with a single HPV infection (86.4%), but three ICC were positive for two HPV types: one contained HPV31 and HPV52, one contained HPV52 and HPV66, and one contained HPV35 and HPV66. HPV16 was the most prevalent type in ICC, accounting for 45.5% of ICC, followed by HPV18 and HPV45, each accounting for 13.6% of ICC. HPV16, -18, and -66 were found more frequently in ICC than in ≤CIN1 (ICC:≤CIN1 ratios 4.61, 1.21, and 1.61, respectively), but HPV66 was only found in ICC with multiple infections, and therefore lost its importance when only single infections were considered. HPV16 and -18, but also HPV31 and -45, were more frequently observed in ICC than in CIN3 (ICC:CIN3 ratios 1.43, 1.52, 1.02, and 1.20, respectively). When adjusted for age, women with ICC were significantly more likely to carry HPV16 compared with women with ≤CIN1 (OR_MH_ 7.36; 95% CI 2.33–23.21); this was also true when only single infections were taken into account.

### 2.3. Methylation Levels Across HPV Type-Specific Disease Categories

**Figure 2:**
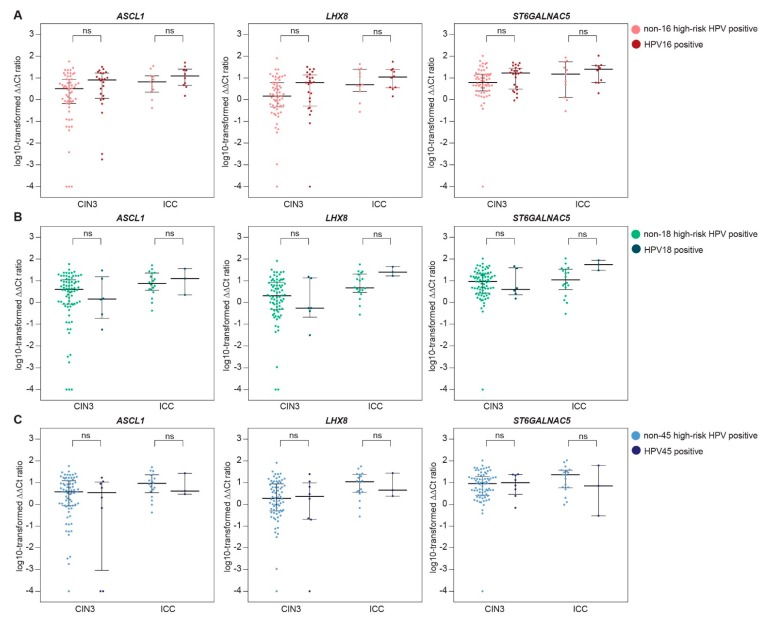
Methylation levels of *ASCL1*, *LHX8*, and *ST6GALNAC5* in cervical scrapes of women with CIN3 and invasive cervical cancer (ICC) associated with HPV16 versus non-16 HPV (**A**), HPV18 versus non-18 HPV (**B**), and HPV45 versus non-45 HPV (**C**). The *y* axis shows the log-transformed ∆∆*C*t ratios. Each dot represents a case and the horizontal lines indicate the medians and interquartile ranges. CIN, cervical intraepithelial neoplasia; HPV, human papillomavirus, ns, not significant.

**Figure 3:**
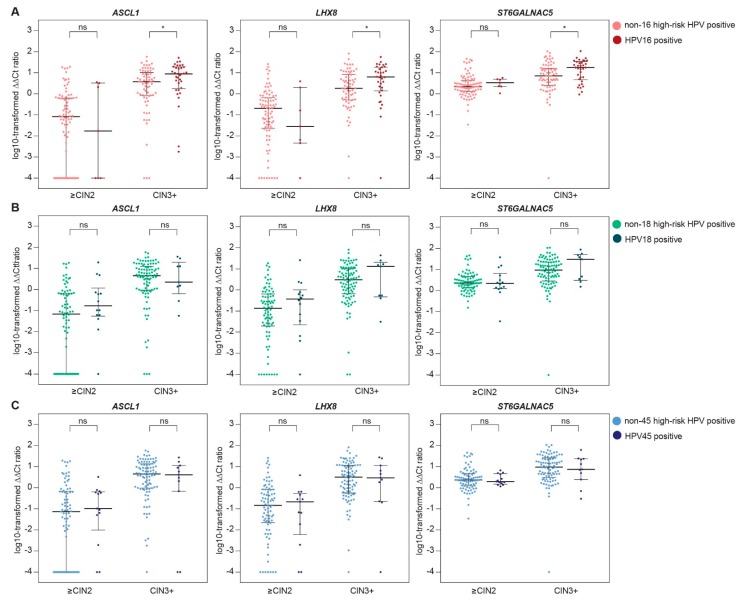
Methylation levels of *ASCL1*, *LHX8*, and *ST6GALNAC5* in cervical scrapes of women with CIN2 or less (≤CIN2) and CIN3 or worse (CIN3+) associated with HPV16 versus non-16 HPV (**A**), HPV18 versus non-18 HPV (**B**), and HPV45 versus non-45 HPV (**C**). The *y* axis shows the log-transformed ∆∆*C*_t_ ratios. Each dot represents a case and the horizontal lines indicate the medians and interquartile ranges. CIN, cervical intraepithelial neoplasia; HPV, human papillomavirus, ns, not significant; * *p*-value < 0.05.

From 197 HPV-positive women, methylation results from a cervical scrape were available. Table 3 shows the comparison of median ∆∆*C*t ratios (i.e., methylation levels) of *ASCL1*, *LHX8*, and *ST6GALNAC5* in cervical scrapes of women with HPV16 versus non-16 HPV, HPV18 versus non-18 HPV, and HPV45 versus non-45 HPV infections, stratified for CIN3 and ICC (Table 3A) and stratified for ≤CIN2 and CIN3+ (Table 3B). A trend towards higher methylation levels in HPV16 positive over non-16 HPV positive was observed in both ICC and CIN3 cases. When analysed together, methylation levels in cervical scrapes of women with CIN3+ associated with HPV16 were significantly higher compared with methylation levels in cervical scrapes of women with CIN3+ associated with other high-risk HPV types. For HPV18, higher methylation levels in HPV18-positive ICC (*n* = 3) over non-18 HPV-positive ICC were observed, and after the exclusion of HPV16-positive cases, this difference reached significance for *LHX8* and *ST6GALNAC5* (*p*-values 0.041 for both markers). This was not observed for HPV18-positive CIN3 lesions. For HPV45, methylation levels in HPV45-positive lesions were comparable to methylation levels in non-45 HPV-positive lesions, also after the exclusion of HPV16-related cases. Figure 2 and Figure 3 show the distribution of the methylation levels of each marker in HPV16 versus non-16 HPV, HPV18 versus non-18 HPV, and HPV45 versus non-45 HPV, stratified for CIN3 and ICC (Figure 2) and stratified for ≤CIN2 and CIN3+ (Figure 3), illustrating the increased methylation levels in HPV16-associated CIN3 and ICC.

## 3. Discussion

Country-based HPV type-specific prevalence ratios across cervical disease categories in WLHIV are needed to make appropriate recommendations on screening and vaccination policies for this high-risk population. In line with previous reports on HPV type-specific prevalence in WLHIV, HPV16 was the most prevalent high-risk type in our study group of WLHIV in South Africa [28]. A steady rise in HPV16 positivity through increasing severity of cervical disease was observed, as opposed to a relatively stable prevalence of other high-risk types. Our finding that women with ICC and CIN3 were significantly more likely to carry HPV16 when compared with women with ≤CIN1 supports that HPV16 confers a preferential risk of developing cervical cancer in WLHIV.

This preferential cancer risk of HPV16 in WLHIV, which has also been described for HIV-uninfected women [29], might be explained by the strong capacity of HPV16 to induce hypermethylation of host cell genes, as reflected by the increased methylation levels in HPV16 over non-16 high-risk HPV-associated CIN3+ observed in this study. Methylation levels of several host cell genes, including *ASCL1*, *LHX8*, and *ST6GALNAC5*, increase with the severity of the underlying cervical disease and are particularly high in cervical scrapes of women with cervical cancer [30,31,32,33,34,35].

So far, a limited number of studies on host cell DNA methylation have included WLHIV [27,31,36,37,38,39]. We previously showed that WLHIV without cervical disease have increased methylation levels of host cell genes compared with HIV-uninfected women without cervical disease [27,31], which might make them more susceptible to HPV-induced cervical neoplasia. It has also been shown that dysregulation of host methylation by HPV16 E6 and E7 by upregulation of DNMT1 expression is associated with host immune suppression during HPV-associated cancer progression [20,40,41,42]. The increased methylation levels of HPV16 compared with non-16 high-risk HPV-associated CIN3+ observed in the present report may reflect a superior ability of HPV16 to upregulate the expression of DNMT1, resulting in increased methylation levels and thereby silencing tumour suppressor genes. To the best of our knowledge, dysregulation of the DNA methylation machinery by other high-risk HPV types has not been described and data on potential type-dependent DNMT deregulation activity are still lacking. At least, our results suggest that host cell methylation levels may be used in monitoring the progression of cervical neoplasms in WLHIV.

It may be speculated that DNMT inhibitors could be effective in the treatment of HPV-induced lesions by reversing the expression of methylation targets such as *ASCL1*, *LHX8*, and *ST6GALNAC5*. However, methylation-dependent expression regulation and the functional role of the genes *ASCL1*, *LHX8*, and *ST6GALNAC5* in cervical carcinogenesis remain to be established. Methylation of *ASCL1*, *LHX8*, and *ST6GALNAC5*, as found in cervical cancer, has also been detected in oral cancer (*ASCL1*) [43], colorectal cancer (*ASCL1* and *ST6GALNAC5*) [44,45], and breast cancer (*LHX8*) [46], suggesting a tumour suppressive role of these genes in these cancers. On the other hand, *ASCL1* and *ST6GALNAC5* have been described as protumorigenic in other cancer types [47,48,49,50].

Some limitations to this study may apply. First, the sample size may have been too small to demonstrate a preferential cancer risk or a specific influence on methylation levels of rare high-risk HPV types. Second, the long-term type-specific cancer risk could not be calculated, as only cross-sectional results were available. Further (prospective) research on the influence of HPV type on host cell methylation levels and the associated cervical cancer risk is needed and should include both HIV-infected and HIV-uninfected women. These studies are in progress.

Our data confirm that although HPV16 is relatively under-represented in WLHIV with cervical cancer, it remains the most important risk factor for ICC. At the same time, the increased prevalence of non-16 high-risk HPV types, most notably HPV45, in cervical carcinomas and CIN3 in WLHIV compared with the general population suggests that specific cervical cancer prevention methods, targeting not only HPV16 but also other high-risk HPV types, are required for this population. Our finding that, in WLHIV, HPV16-associated CIN3 lesions and cervical cancer have increased methylation levels compared with lesions associated with other HPV types requires further exploration and advocates further research into screening algorithms that combine HPV genotyping with methylation analysis.

## 4. Methods

### 4.1. Study Population and Specimen Collection

Between February 2013 and March 2015, women aged 18 years and above who had not been treated for cervical cancer or precancer in the preceding two years were recruited from a gynaecological outpatient clinic in Steve Biko Academic Hospital and Tshwane District Hospital, Pretoria, South Africa, as part of a study comparing different cervical screening strategies. The the Faculty of Health Sciences Research Ethics Committee of the University of Pretoria approved the study (protocol numbers 100/2012 and 155/2014, 25 September 2012 and 18 June 2014). Written informed consent was obtained from all participants.

In the present report, HIV-seropositive women from two study groups were included (see Figure 1): 355 women from a screening cohort of WLHIV and 122 women from a gynaecology referral population. From 422 participants, a cervical scrape was collected as described previously [31]. From 55 women from the gynaecological referral population, a self-collected cervical specimen was available. Women included in the screening cohort visited the gynaecologic outpatient clinic for cervical screening. A cervical sample was collected using a Cervex Brush^®^ (Rovers Medical Devices B.V., Oss, The Netherlands) and, after the preparation of a conventional slide, the cells were stored in Thinprep PreservCyt^®^ (Hologic, Marlborough, MA, USA). Colposcopy was then performed in all participants and two mandatory biopsies were taken. Women included in the referral population were seen at the gynaecologic outpatient department for evaluation of an abnormal Pap smear (≥high-grade squamous intraepithelial neoplasia (HSIL), *n* = 104) or a histologically proven cervical carcinoma (*n* = 18). Prior to gynaecological examination, a cervical specimen was collected by either the physician using a Cervix brush (*n* = 67), or by the participant herself using a Delphi Screener (Delphi Bioscience B.V., Scherpenzeel, The Netherlands, *n* = 55), and the cellular material was stored in Thinprep PreservCyt solution.

Clinical data including HIV status, most recent CD4+ count, and the use of antiretroviral treatment (ART) were recorded. All histology specimens were classified as either no dysplasia, CIN1, CIN2, CIN3, or invasive cervical cancer (ICC) according to international criteria [51]. Women with abnormal cytology (≥HSIL) or CIN2 or worse (CIN2+) on a cervical biopsy were treated according to local guidelines (large loop excision of the transformation zone (LLETZ) or clinical staging). The most severe histological diagnosis, based on either the biopsy or the LLETZ specimen, was used as the study endpoint. Women without valid study endpoints were excluded from the analyses.

### 4.2. High-Risk HPV Testing

Molecular analyses were performed at the Department of Pathology of Amsterdam UMC, Vrije Universiteit Amsterdam, Amsterdam, The Netherlands. Nucleic acids were isolated from physician-taken and self-collected cervical specimens using the Nucleo-Spin 96 Tissue kit (Macherey-Nagel GmbH & Co., KG, Düren, Germany) and a Microlab STAR robotic system (Hamilton Company, Reno, Nevada, USA) according to the manufacturer’s instructions. DNA isolates were tested for the presence of high-risk HPV DNA using the clinically validated GP5+/6+ PCR enzyme immunoassay (EIA) as described previously [52]. Separate β-globin polymerase chain reaction (PCR) analysis was conducted for sample quality control. Samples testing negative for high-risk HPV and β-globin were considered invalid. Genotyping of EIA-positive samples was performed using the HPV-risk assay (Self-screen B.V., Amsterdam, The Netherlands), which can differentiate between HPV16, -18, and other high-risk HPV types [53,54], and/or a bead-based analysis of GP5+/6+ PCR products, which can fully differentiate 14 high-risk types (16, 18, 31, 33, 35, 39, 45, 51, 52, 56, 58, 59, 66, and 68) [55]. EIA-positive samples without a specific genotype result from the abovementioned assays are referred to as “HPV-X”.

### 4.3. Methylation Analysis

For methylation analysis, DNA isolated from cervical scrapes was used. Multiplex quantitative methylation-specific PCR (qMSP) for *ASCL1*, *LHX8*, and *ST6GALNAC5* was performed using 50 ng of bisulphite-converted DNA, as described previously [27]. Sample quality and successful bisulphite conversion was assured using the housekeeping gene B-actin (ACTB) as a reference. All samples with ACTB *C*t ratios >30 were excluded from the analysis. The comparative *C*t method (2^−∆∆*C*t^ × 100), which normalises methylation values of all targets to the reference gene and the calibrator, was used to obtain ∆∆*C*_t_ ratios [56].

### 4.4. Statistical Analysis

Overall HPV prevalence was assessed within disease categories based on histology (i.e., no dysplasia or CIN1 (≤CIN1), CIN2, CIN3, and ICC). Type-specific positivity is reported as the proportion of HPV-positive cases in which the particular HPV type was detected. Differences in type-specific prevalence for women with CIN3 compared with women without evidence of high-grade cervical disease (CIN3:≤CIN1), and women with ICC compared with women with ≤CIN1 (ICC:≤CIN1), were examined using prevalence ratios. The same was done to compare type-specific prevalence in women with ICC to type-specific prevalence in women with CIN3 (ICC:CIN3). The Mantel–Haenszel common odds ratios (OR_MH_) and 95% confidence intervals (95% CI) were calculated to adjust for age. Only OR_MH_ values of 1.0 and higher are reported. Data were adjusted for 10-year age categories (i.e., below 30 years, 30–39, 40–49, 50–59, and 60 years and over). Breslow–Day’s test of homogeneity was used to determine the presence of an association between OR_MH_ and age. Analyses were performed separately for women with any HPV infection (single and multiple infections combined) and for women with single infections only.

Differences in methylation levels in cervical scrapes were compared between HPV types (HPV16 versus other, HPV18 versus other, and HPV45 versus other) in women with and without CIN3 or worse (CIN3+ and ≤CIN2, respectively) using Mann–Whitney U tests. *p*-values of <0.05 were considered significant. Since HPV16 infections heavily dominated in ICC cases, we also performed the analysis for ICC and CIN3 cases separately, and for HPV18 and HPV45 the analyses were also performed after discarding HPV16-positive cases.

## Figures and Tables

**Table 1:** HPV prevalence within disease categories.

	HPV-Negative		HPV-Positive					
			Any *		Single		Multiple	
	*n*	%	*n*	%	*n*	%	*n*	%
≤CIN1 (*n* = 254)	183	72.0%	71	28.0%	55	77.5%	16	22.5%
CIN2 (*n* = 54)	12	22.2%	42	77.8%	23	54.8%	19	45.2%
CIN3 (*n* = 133)	10	7.5%	123	92.5%	83	67.5%	40	32.5%
ICC (*n* = 22)	0	0.0%	22	100%	19	86.4%	3	13.6%

CIN, cervical intraepithelial neoplasia; ICC, invasive cervical cancer; HPV, human papillomavirus. * Includes both multiple and single infections.

**Table 2:** HPV type-specific prevalence across disease categories among HPV-positive women—overall and for single infections separately.

	≤CIN1 *n* = 71		CIN2 *n* = 42		CIN3 *n* = 123		ICC *n* = 22		Total *n* = 258		CIN3:≤CIN1	ICC:≤CIN1	ICC:CIN3	CIN3 vs. ≤CIN1		ICC vs. ≤CIN1		ICC vs. CIN3	
Type	*n*	%	*n*	%	*n*	%	*n*	%	*N*	%	PR	PR	PR	OR (95% CI)		OR (95% CI)		OR (95% CI)	
*Overall* *																			
16	7	9.9%	6	14.3%	39	31.7%	10	45.5%	62	24.0%	3.22	4.61	1.43	**4.37**	**(1.81–10.58)**	**7.36**	**(2.33–23.21)**	1.62	(0.62–4.27)
18	8	11.3%	8	19.0%	11	8.9%	3	13.6%	30	11.6%	0.79	1.21	1.52	-		1.10	(0.25–4.79)	1.83	(0.38–8.75)
31	7	9.9%	7	16.7%	11	8.9%	2	9.1%	27	10.5%	0.91	0.92	1.02	-		1.04	(0.19–5.59)	1.37	(0.26–7.15)
33	5	7.0%	3	7.1%	14	11.4%	1	4.5%	23	8.9%	1.62	0.65	0.40	1.94	(0.34–5.89)	-		-	
35	6	8.5%	9	21.4%	14	11.4%	1	4.5%	30	11.6%	1.35	0.54	0.40	1.83	(0.65–5.15)	-		-	
39	2	2.8%	1	2.4%	2	1.6%	-	-	5	1.9%	0.58	-	-	-		-		-	
45	10	14.1%	6	14.3%	14	11.4%	3	13.6%	33	12.8%	0.81	0.97	1.20	-		-		1.25	(0.33–4.78)
51	1	1.4%	5	11.9%	5	4.1%	-	-	11	4.3%	2.89	-	-	2.88	(0.31–27.06)	-		-	
52	12	16.9%	5	11.9%	17	13.8%	2	9.1%	36	14.0%	0.82	0.54	0.66	-		-		-	
56	10	14.1%	4	9.5%	11	8.9%	1	4.5%	26	10.1%	0.63	0.32	0.51	-		-		-	
58	3	4.2%	4	9.5%	14	11.4%	-	-	21	8.1%	2.69	-	-	2.73	(0.75–9.95)	-		-	
59	4	5.6%	3	7.1%	5	4.1%	-	-	12	4.7%	0.72	-	-	-		-		-	
66	4	5.6%	3	7.1%	14	11.4%	2	9.1%	23	8.9%	2.02	1.61	0.80	1.96	(0.61–6.35)	1.63	(0.24–11.07)	-	
68	-	-	-	-	-	-	-	-	-	-	-	-	-	-		-		-	
X	9	12.7%	4	9.5%	11	8.9%	-	-	24	9.3%	0.71	-	-	-		-		-	
*Single*	*n* = 55		*n* = 23		*n* = 83		*n* = 19		*n* = 180										
16	4	7.3%	1	4.3%	20	24.1%	10	52.6%	35	7.6%	3.31	7.24	2.18	**3.79**	**(1.19–12.09)**	**11.4**	**(3.10–41.90)**	**3.01**	**(1.06–8.55)**
18	7	12.7%	3	13.0%	4	4.8%	3	15.8%	17	3.7%	0.38	1.24	3.28	-		1.02	(0.21–4.90)	4.36	(0.56–34.0)
31	4	7.3%	2	8.7%	4	4.8%	1	5.3%	11	2.4%	0.66	0.72	1.09	-		-		1.03	(0.09–12.31)
33	2	3.6%	-	-	7	8.4%	1	5.3%	10	2.2%	2.32	1.45	0.62	2.85	(0.54–15.08)	1.25	(0.09–17.45)	-	
35	2	3.6%	6	26.1%	8	9.6%	0	-	16	3.5%	2.65	-	-	3.78	(0.74–19.32)	-		-	
39	1	1.8%	-	-	1	1.2%	0	-	2	0.4%	0.66	-	-	-		-		-	
45	7	12.7%	1	4.3%	5	6.0%	3	15.8%	16	3.5%	0.47	1.24	2.62	-		1.11	(0.26–4.65)	2.58	(0.54–12.42)
51	1	1.8%	1	4.3%	2	2.4%	0	-	4	0.9%	1.33	-	-	2.00	(0.17–23.43)	-		-	
52	6	10.9%	2	8.7%	7	8.4%	0	-	15	3.2%	0.77	-	-	-		-		-	
56	6	10.9%	-	-	5	6.0%	1	5.3%	12	2.6%	0.55	0.48	0.87	-		-		1.56	(0.17–14.15)
58	1	1.8%	1	4.3%	6	7.2%	0	-	8	1.7%	3.98	-	-	4.30	(0.50–36.68)	-		-	
59	2	3.6%	1	4.3%	1	1.2%	0	-	4	0.9%	0.33	-	-	-		-		-	
66	3	5.5%	1	4.3%	3	3.6%	0	-	7	1.5%	0.66	-	-	-		-		-	

Only Mantel–Haenszel common odds ratios (OR_MH_) with values of 1.0 or higher are reported. Data are adjusted into 10-year age categories (below 30 years, 30–39, 40–49, 50–59, and 60 years and older). Significant odds ratios are depicted in bold typeface. * Multiple and single infections combined. CIN, cervical intraepithelial carcinoma; ICC, invasive cervical cancer; PR, prevalence ratio.

**Table 3:** Comparison of median ∆∆*C*t ratios of *ASCL1*, *LHX8*, and *ST6GALNAC5* between HPV types within CIN3 and invasive cervical cancer (ICC) (**A**), and within ≤CIN2 and CIN3+ (**B**).

**A.**				**ASCL1**		**LHX8**		**ST6GALNAC5**	
			***N***	**Median**	***p*** **-Value**	**Median**	***p*** **-Value**	**Median**	***p*-Value**
*HPV16* vs. *other types*									
	CIN3	other	58	3.21		1.46		6.27	
		HPV16	24	8.14	0.09	6.16	0.08	16.68	0.05
	ICC	other	11	6.54		4.86		15.16	
		HPV16	10	12.69	0.26	11.05	0.73	25.48	0.53
*HPV18* vs. *other types* *									
	CIN3	other	76	4.03		2.04		9.09	
		HPV18	6	1.45	0.71	0.55	0.55	4.02	0.78
	ICC	other	18	7.58		4.68		11.55	
		HPV18	3	12.56	0.62	24.84	0.07	54.85	0.06
*HPV45* vs. *other types* *									
	CIN3	other	74	3.82		1.90		8.92	
		HPV45	8	3.91	0.60	2.39	0.76	10.32	0.90
	ICC	other	18	9.22		10.98		22.86	
		HPV45	3	4.07	0.76	4.49	0.92	7.11	0.62
**B.**				**ASCL1**		**LHX8**		**ST6GALNAC5**	
			**N**	**Median**	***p*-Value**	**Median**	***p*-Value**	**Median**	***p*-Value**
*HPV16* vs. *other types*									
	≤CIN2	other	87	0.08	-	0.20	-	2.17	-
		HPV16	7	0.02	0.90	0.03	0.50	3.29	0.34
	CIN3+	other	69	3.75	-	1.83	-	7.11	-
		HPV16	34	8.83	0.017	6.34	0.019	17.79	0.026
*HPV18* vs. *other types* *									
	≤CIN2	other	80	0.07	-	0.13	-	2.26	-
		HPV18	14	0.18	0.10	0.37	0.34	2.17	0.87
	CIN3+	other	94	4.48	-	3.03	-	9.09	-
		HPV18	9	2.24	0.88	13.08	0.48	29.92	0.27
*HPV45* vs. *other types* *									
	≤CIN2	other	81	0.07	-	0.14	-	2.37	-
		HPV45	13	0.10	0.58	0.21	0.67	1.94	0.93
	CIN3+	other	92	4.48	-	3.20	-	9.56	-
		HPV45	11	4.07	0.69	2.97	0.94	7.49	0.81

* Results were similar when HPV16-positive cases were excluded. CIN, cervical intraepithelial neoplasia; ICC, invasive cervical cancer; HPV, human papillomavirus.
